# Long-Term Survival in Human Epidermal Growth Factor Receptor 2-Positive Bone-Only Metastatic Breast Cancer: Trastuzumab, Denosumab, and Potential Synergistic Effects

**DOI:** 10.7759/cureus.68576

**Published:** 2024-09-03

**Authors:** Rahman Ladak, Bre-Anne Fifield, Lisa Porter, Khalid Hirmiz, Caroline Hamm

**Affiliations:** 1 Schulich School of Medicine and Dentistry, Western University, London, CAN; 2 Department of Biomedical Sciences, University of Windsor, Windsor, CAN; 3 Department of Radiation Oncology, Windsor Regional Hospital, Windsor, CAN; 4 Department of Medical Oncology, Windsor Regional Hospital, Windsor, CAN

**Keywords:** synergistic effects, denosumab, trastuzumab, metastatic breast cacner, rank and rankl, case report series, progression - free survival, human epidermal growth factor receptor 2 (her2), her2-positive breast cancer

## Abstract

Breast cancer is the second most common cancer worldwide. There are four main subtypes of breast cancer, one of which involves positivity for human epidermal growth factor receptor 2 (HER2). Here, we present a case series of unusually long survival in three patients with HER2-positive metastatic breast cancer. All cases involved post-menopausal women with bone-only metastases undergoing treatment with the HER2-targeted therapy trastuzumab and the receptor activator of nuclear factor kappa-Β ligand (RANK-L) inhibitor denosumab. Our three patients survived for 17, 13, and 11 years, respectively, from the time of metastasis. The patients who survived for 17 and 13 years both presented with metastatic disease at diagnosis, while the patient who survived for 11 years with metastatic disease was known to have non-metastatic breast cancer for four years prior. We also report the development of foot fractures from minor trauma, as low as walking, despite a bone density reported as normal in the patient with 17 years of treatment. These unusually long survival times and the unusual location of the fractures are questioned to be secondary to the long duration of treatment with HER2-targeted therapy and RANK-L inhibitor therapy. Our case series is the first to describe the use of trastuzumab and denosumab in HER2-positive metastatic breast cancer. All three reported cases had no clinical or radiographic disease progression at the time of reporting. Furthermore, our case of survival for 17 years represents the longest survival time reported yet, raising the possibility of a synergistic relationship between RANK-L inhibitors and HER2-targeted therapy in the long-term control of HER2-positive metastatic breast cancer. This manuscript discusses evidence from primary studies on HER2 and receptor activator of nuclear factor kappa-Β (RANK) signalling and drug responses and hypothesizes on possible mechanisms of synergism. Given that treatment of HER2-positive breast cancer has historically not involved RANK-L inhibition, this study may outline future areas of research in improving treatment algorithms, especially for bone-only metastatic disease.

## Introduction

Human epidermal growth factor receptor 2 (HER2), which is a membrane tyrosine kinase receptor and oncogene, is expressed at high levels in 15-20% of breast tumours [1]. It is also sometimes known as erythroblastic oncogene B2 (ERBB2) and CD340. Alongside oestrogen receptors (ER), HER2 and ER are the known dominant drivers of cell proliferation and survival in 85% of breast cancers [1]. HER2 enrichment in breast cancer tumours is correlated with greater rates of metastasis [2]. In 3-6% of cases, patients present with metastatic disease at diagnosis (de novo metastasis) [3]. The HER2-targeted therapy trastuzumab has improved survival for patients diagnosed with HER2-positive breast cancer, and several case reports have even described long-term survival in patients with HER2-positive metastatic breast cancer (MBC) [4-8].

This case series describes unusually long survival in three patients with HER2-positive bone-only MBC. All three patients were treated with trastuzumab and the receptor activator of nuclear factor kappa-Β ligand (RANK-L) inhibitor denosumab. The patients survived for 17, 13, and 11 years, respectively, with no recurrence of their primary tumour and little progression of their bone metastases. These ongoing progression-free survival timelines are significantly higher than the average survival of 40-65 months [9], the five-year overall survival rate of 7% [2], and the longest previously reported survival time of 14 years with HER2-positive MBC [7]. Moreover, our cases are unique because none of our patients have had disease progression radiographically or clinically at the time of reporting, implying a longer overall survival.

This raises the possibility of a potential synergistic mechanism between HER2-targeted and RANK-L inhibitor therapies. Also reported in our longest-surviving patient is the development of foot fractures from minor trauma as minimal as walking, despite a bone density reported as normal. The unusual location of the fractures was questioned to be secondary to the long duration of treatment with HER2-targeted and RANK-L inhibitor therapies.

## Case presentation

Methodology

Patients were selected from a large community cancer center that sees, on average, 400 breast cancer patients per year. Over a period of 20 years, the patients who met the following characteristics were identified by treating physicians: long-term disease-free survival, HER2-positive cancer, any HER2-directed therapy, any bone-directed therapy, and any endocrine therapy. Coincidentally, the three patients who met all the above criteria were all treated with the same HER2-directed and bone-directed therapies of trastuzumab and denosumab. All patients had biopsy-proven metastatic disease, as outlined in Table 1.

**Table 1:** Demographics and pathological characteristics of patients with HER2-positive metastatic breast cancer in this case series.

Patient	1	2	3
Hormone receptor histopathology	ER+ 70%/PR+ 15%, Grade 3	ER+ 15%/PR 0%, Grade 3	ER+ 60%/PR 0%, Grade 3
Age at presentation of breast cancer	52	68	41
Period of ongoing metastatic disease stability at time of reporting	17	13	11
Stage of disease at diagnosis	Stage IV with unifocal bony sclerotic metastases to sternum	Stage IV with bony metastases to the skull, ribs, and pelvis	Inflammatory breast cancer; progressed to stage IV with bony metastasis at T11, four years after initial presentation
Chemotherapy regimen	Doxorubicin/cyclophosphamide/paclitaxel-trastuzumab (AC-TH regimen)	Paclitaxel-trastuzumab	Doxorubicin/cyclophosphamide/paclitaxel-trastuzumab (AC-TH regimen)
Surgical treatment	Bilateral mastectomy	None	Unilateral mastectomy
Radiotherapy	Radiation dose to sternum	None	Adjuvant radiotherapy only
HER2-directed therapy	Trastuzumab	Trastuzumab	Trastuzumab
Bone-directed therapy	Denosumab	Pamidronate (5 months total) changed to denosumab for tolerability	Denosumab
Endocrine therapy	Anastrozole changed to tamoxifen for tolerance	Letrozole	Oophorectomy, letrozole changed to fulvestrant for tolerance
Adverse events	Metatarsal factures 15 years into treatment, appearing to be due to hypophosphatasia	Atrial fibrillation 6 years into treatment, treated with apixaban, carvedilol, lisinopril (possibly age-related); cardiac toxicity 9 years into treatment involving reduced cardiac ejection fraction; remained on trastuzumab under the supervision of cardiology	None

Case 1

A postmenopausal 52-year-old female presented with immunohistochemistry (IHC) HER2-positive, 70% ER-positive, and 15% progesterone receptor (PR)-positive MBC in 2007 and has been followed for 17 years with ongoing stable disease. Before her diagnosis, the patient presented with several months of sternal pain. A bone scan from April 2007 revealed a large area of intense uptake in the body of the sternum from the sterno-manubrial junction to two-thirds of the distance to the xiphoid. CT scans did not demonstrate any other definitive metastatic disease. She had an initial lumpectomy, demonstrating a 1.3 cm tumor that was 70% ER-positive, 15% PR-positive, equivocal by IHC for HER2, and positive for HER2 by fluorescence in situ hybridization (FISH). Following dose-dense doxorubicin, cyclophosphamide, paclitaxel, and trastuzumab chemotherapy, she underwent a bilateral mastectomy procedure and right axillary lymph node dissection. Pathology revealed three out of the eight resected lymph nodes were positive for tumor cells. Thereafter, she underwent 25 fractions of radiotherapy to the sternal lesion. She continued trastuzumab, anastrozole, and started denosumab in September 2007, and continues these medications at the time of reporting. Denosumab was administered every four weeks until 2013 and then every twelve weeks until 2020. Since 2020, denosumab has been administered every six months. Likewise, the frequency of trastuzumab administration was changed from every three weeks until 2017 to every six weeks until 2022 and thereafter to every three months. The patient's cardiac function remained stable, with an ejection fraction (EF) greater than 50% on routine monitoring. No progression of her cancer has been detected to date with routine imaging.

**Figure 1:**
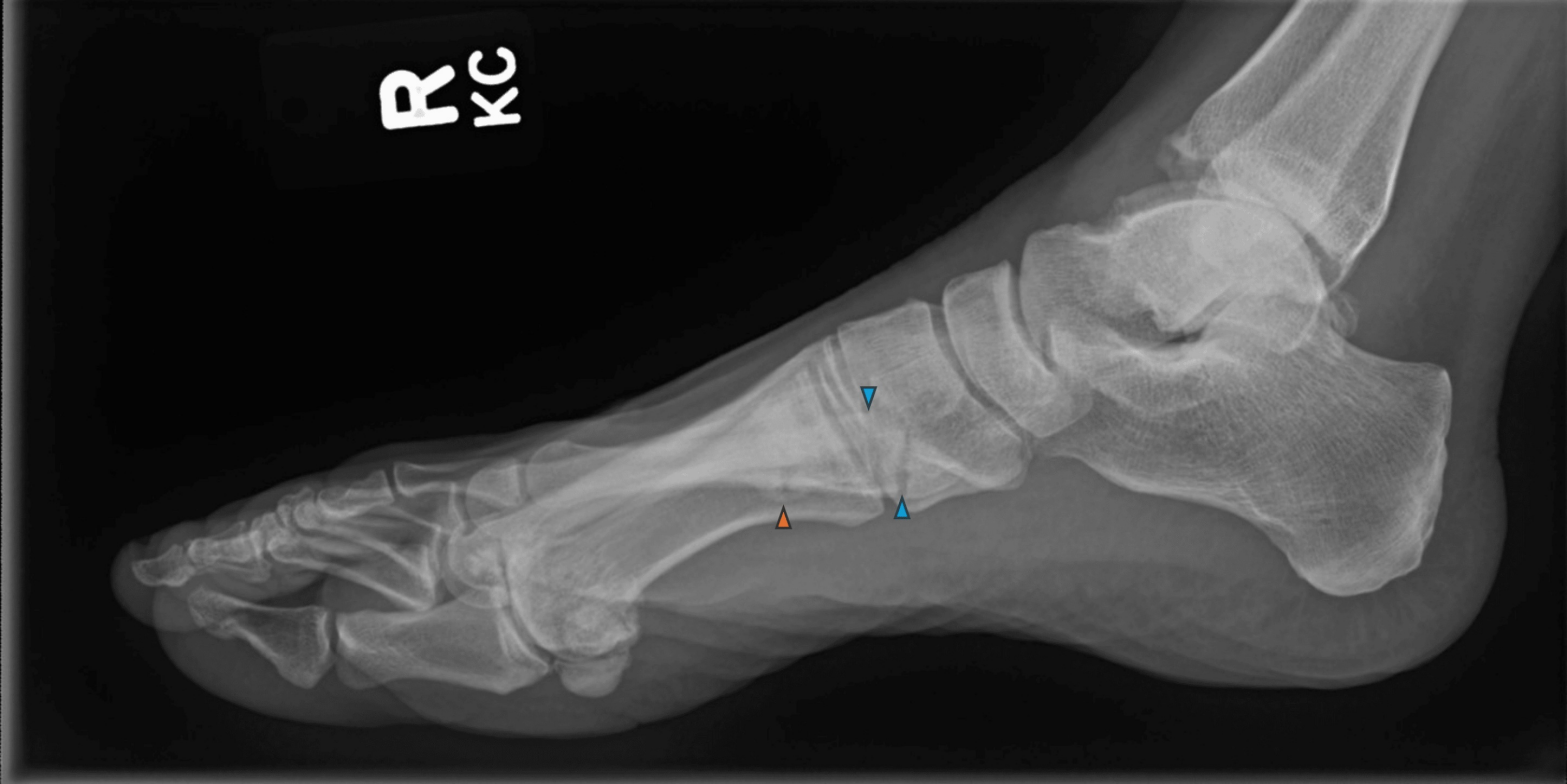
Radiograph demonstrating normal alignment and mineralization of metatarsals in case 1. There is a non-displaced fracture of the base of the fifth metatarsal bone with two fracture lines visible (blue arrowheads), and no definite intra-articular extension. An old non-displaced healed fracture of the proximal meta-diaphysis of the fourth metatarsal bone with sclerosis at the fracture margin and callus formation are also visualized (orange arrowhead).

After 15 years of treatment, the patient began suffering from metatarsal fractures following minor trauma as low as walking. Investigations confirmed the patient’s normal bone density (T +1.6, +0.3, and +0.2). However, considering the odd location of the fractures and the anti-resorptive medications (denosumab and tamoxifen) that the patient was receiving, she was referred to an endocrinologist and bone disease specialist, who confirmed that the patient did not have any clinical features of osteogenesis imperfecta or osteoporosis. Lab tests showed that the patient’s calcium levels were within normal limits. However, her alkaline phosphatase levels were low (31-34 IU/L), in keeping with hypophosphatasia. Likewise, imaging of the fractures (Figure 1) did not reveal any mineralization or alignment defects. No causal link was made to her medications. After extending her trastuzumab every three months, she recovered from her fractures and had no further fractures.

Case 2

A 61-year-old female presented with progressive nipple inversion in 2011. A breast MRI revealed a 2.7 cm mass at 1 o'clock in the right breast, as well as a possible 1.1 cm metastatic lymph node. The mass was biopsied in April 2011, and pathology revealed an invasive and in-situ mammary carcinoma. A fine needle aspiration biopsy of the adjacent lymph node was also positive. Her tumor was found to be 95% HER2-positive by IHC, 15% ER-positive, and PR-negative. A bone scan conducted at the same time identified multiple bony metastases in the right frontal region of the skull, several ribs bilaterally, and the pelvis bilaterally. A CT scan of the abdomen was negative for metastatic disease. The patient was treated with paclitaxel and trastuzumab between June and September 2011, as well as pamidronate at this time. In October 2011, she was changed from pamidronate to denosumab because of intolerance, was started on anastrozole, and continued trastuzumab throughout. Since starting, she has received denosumab every four weeks.

Regular follow-up imaging has confirmed ongoing stable metastatic disease. During regular monitoring for trastuzumab-related cardiotoxicity, it was noted that her EF dropped from 50-60% in 2020 to 45-50% in 2021. A multigated acquisition scan revealed mild global hypokinesis in 2022 with a stable EF of 44%, but no further decline was noted, and a repeat echo in 2023 showed her EF recover to 50%. With cardiology's support, she was continued on trastuzumab throughout, using an angiotensin-converting enzyme inhibitor and a beta blocker. She has developed atrial fibrillation since 2017, which was not thought to be related to her treatment.

Case 3

A 41-year-old female presented in November 2009 with inflammatory breast cancer. A core biopsy was equivocal for HER2-positivity by IHC but positive for HER2 by FISH. The tumor was also 60% ER-positive and PR-negative. The patient received neoadjuvant dose-dense doxorubicin and cyclophosphamide, followed by paclitaxel and trastuzumab. In March 2020, she underwent a mastectomy and axillary lymph node dissection with immediate reconstruction. This procedure confirmed a 1.0 cm mass with dermal lymphatic invasion. The pathology of this mass confirmed it to be the residual tumor of invasive ductal carcinoma, with one out of the three sampled lymph nodes being positive for tumor cells. Thereafter, the patient received 25 fractions of radiotherapy and tamoxifen, adjuvant zoledronic acid, and goserelin. She completed one year of adjuvant trastuzumab.

In February 2013, four years after her initial diagnosis, her breast cancer metastasized to the T11 vertebral body. Repeat staging identified this as an isolated metastatic site. A biopsy of the T11 lesion revealed a poorly differentiated adenocarcinoma that was ER-positive and PR-negative. HER2 was not retested at that time. Following her biopsy, the patient’s endocrine therapy was switched to letrozole, which was poorly tolerated, then to fulvestrant, which she continues to receive. At this time, she also started denosumab, receiving it every four weeks, and restarted her trastuzumab. She remains on both denosumab and trastuzumab. Regular imaging continues to demonstrate stable metastatic disease. Cardiac screening continues to demonstrate a stable EF between 55% and 65%.

**Figure 2:**
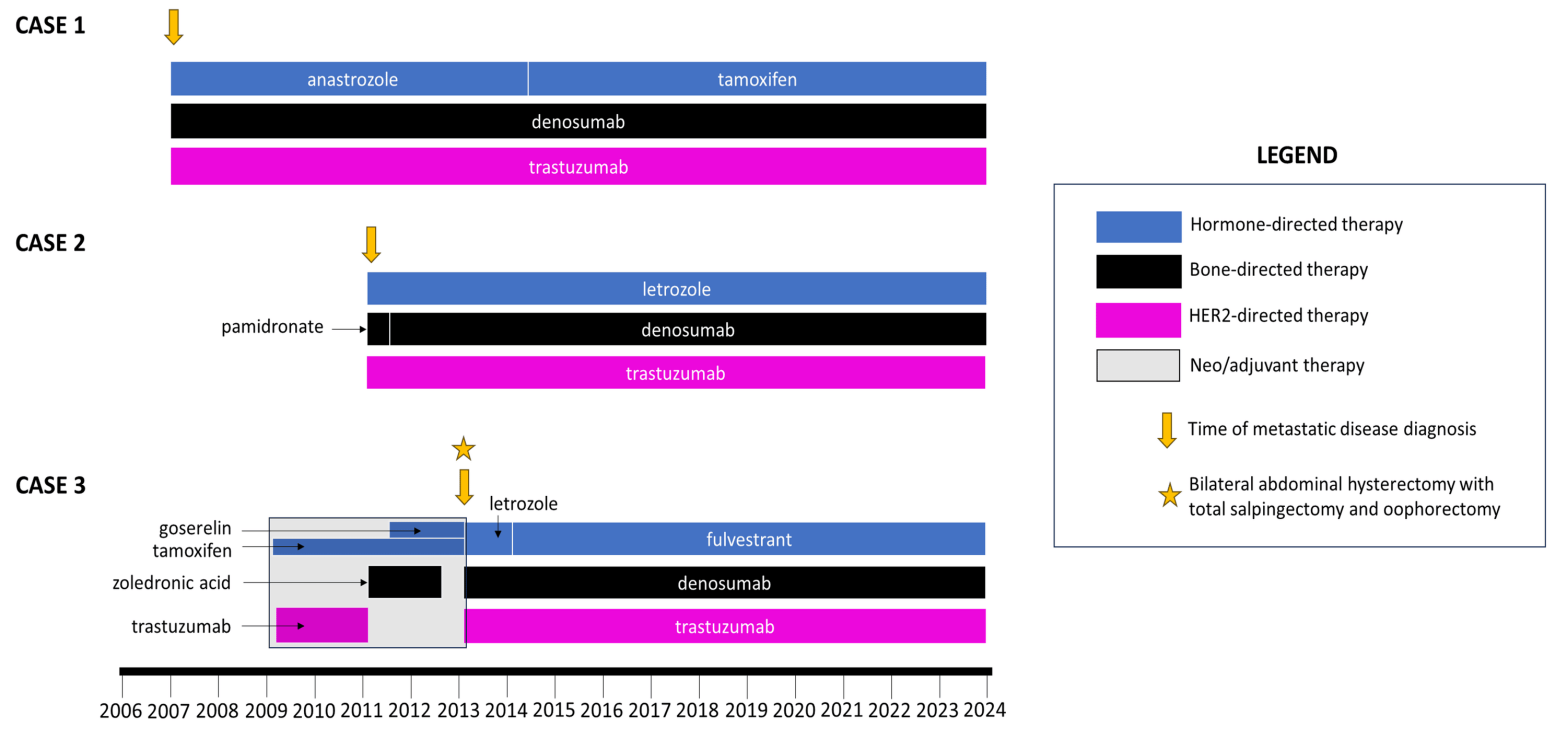
Treatment timelines for the three patients with HER2-positive metastatic breast cancer discussed in this case series.

A timeline summarizing the treatments for all three patients is shown below in Figure 2. The timeline demonstrates the continuity of treatment with each patient's hormone-directed therapy, bone-directed therapy, and HER2-directed therapy.

## Discussion

The three presented cases demonstrate long-term progression-free survival in patients with metastatic HER2-positive breast cancer. HER2-positive MBC has an average survival time of 40-65 months [9] and a five-year overall survival rate of 7% [7], and HER2-positive breast cancer that is metastatic at diagnosis (de novo metastasis) usually has a three-year survival rate of 50-70% [10]. However, case reports published in the literature have suggested that a fraction of patients with HER2-positive MBC may be exceptional responders who can survive for more than 10 years [11]. To confirm this, we performed a literature search on PubMed for “HER2, metastatic breast cancer, case, long-term survival, trastuzumab” and found the case reports outlined in Table 2.

**Table 2:** Previously reported cases of long-term survival with HER2-positive metastatic breast cancer for 10 or more years.

Case number and citation	ER+ and PR+ status	Location of metastases	Treatment	Survival time post-metastasis in years	Patient outcome at the time of publication
1 [4]	−	Brain, leptomeningeal carcinomatosis	Chemotherapy, mastectomy, trastuzumab, lapatinib	12	Progression of disease
2 [5]	+	Lung, lymph node, bone	Chemotherapy, mastectomy, trastuzumab, hormonal therapy	10	Pleural diffusion, stopped trastuzumab
3 [6]	−	Brainstem	Surgical resection of brainstem metastasis, hormonal therapy, trastuzumab	10	No radiographic or clinical evidence of recurrent disease
4 [7]	−	Lung, liver	Chemotherapy, mastectomy, trastuzumab	14	Minimal significant adverse effects
5 [8]	Not mentioned	Liver	Mastectomy, trastuzumab, chemo-endocrine therapy	11	Progressive remission

Compared to the above case reports, our case series is unique because all three of our patients have ongoing progression-free survival. There has been no evidence of disease progression, clinically or radiographically, and all three patients are continuing their treatments with stable cardiac status and minimal side effects. Moreover, the longest reported survival for patients with similarly presenting disease (post-menopausal HER2-positive MBC) was 14 years [7], making our case with 17 years of progression-free survival a new record. These differences may be attributed to unique features in our cases when compared with previous case reports and reported series. Most importantly, all our patients had bone-only metastatic disease, while previously reported cases involved metastases to the liver, lungs, lymph nodes, and brain. This is important because bone-only MBC demonstrates improved overall survival than breast cancer with visceral metastases [10,11].

Additionally, our three patients’ breast cancers all had high-risk cancer pathologies according to metastatic prognostic staging systems [10]. Previously reported cases of long-term survival with HER2-positive MBC typically involved hormone receptor-negative tumors, in contrast to our patients, who were all ER-positive. The prognostic scoring system proposed by Plichta et al. would suggest that the high-grade tumors and PR-negativity of our patients would place them in higher-risk groups [10]. Nevertheless, our patients' long-term survival despite their higher-risk pathology raises the question of whether a unique synergistic effect may have played a role.

Physiologic and oncologic roles of the receptor activator of nuclear factor kappa-β

The delicate balance in bone remodelling by osteoblasts and osteoclasts is in part mediated by the receptor activator of nuclear factor kappa-Β (RANK) and its ligand RANK-L. RANK is a member of the tumour necrosis factor (TNF) superfamily and regulates osteoclast development. The RANK-L decoy receptor osteoprotegerin competitively inhibits RANK-L, thereby reducing RANK receptor activation, osteoclast maturation, and, ultimately, bone resorption. However, osteoprotegerin expression is controlled by oestrogen, which is decreased in post-menopausal women. This leads to increased RANK signalling, bone resorption, and osteoporosis as osteoprotegerin expression decreases [12]. Similarly, the RANK receptor-ligand pair is involved in mammary epithelial cell proliferation for breast tissue development [13]. This signalling is necessary for lactation but also plays a large role in breast carcinogenesis since mammary epithelial cells are enriched in RANK receptors [13,14].

The role of RANK in HER2-positive breast cancer metastasis

All three of our patients’ HER2-targeted therapy was accompanied by denosumab, a RANK-L inhibitor that prevents bone resorption. This raises the possibility of a unique synergistic effect responsible for the longer survival times of our patients, especially in the context of HER2-positive bone-only MBC.

The SOLTI-1114 PAMELA study by Sanz-Moreno et al. demonstrated RANK and RANK-L were more frequently detected in HER2-positive tumours that acquired resistance to anti-HER2 therapies than in treatment-naive ones [15]. This finding raised the question of whether the RANK receptor-ligand pair is involved in resistance to HER2-directed therapies. Sanz-Moreno et al. demonstrated a direct interaction between the RANK receptor and HER2 using a proximity ligation assay. Their results supported the hypothesis that pro-tumour signalling through RANK/HER2 heterodimers could be responsible for resistance to HER2-therapy [15]. This was further supported in a study by Zoi et al., which presented evidence that combining trastuzumab with denosumab in HER2-positive breast cancer cell lines diminished HER2/RANK heterodimerization more efficiently than single targeting [16]. These alterations are associated with reduced invasiveness of breast cancer cells and improved clinical outcomes [16]. Zhang et al. outlined how denosumab inhibits HER2/RANK heterodimer activation and signalling, possibly improving the response to anti-HER2 therapies in RANK-positive HER2-positive breast cancer patients. They also conducted a follow-up study confirming that RANK-L decreases the efficacy of HER2 inhibitors. In this study, all patients received trastuzumab and lapatinib. Zhang et al. also suggested that increased RANK signalling may contribute to the development of lapatinib resistance through nuclear factor kappa-Β (NF-κΒ) activation [17].

In an unselected group of breast cancer patients, a multivariate survival analysis (cox regression) conducted by Papanastasiou et al. indicated that the worse clinical outcome observed for the EGFRhi/RANKhi group was independent of stage and primary lymph node status (p = 0.008) [18]. Although Papanastasiou’s study did not report if any of the patients received primary RANK-L inhibitor treatment, their results suggest that they were identifying patients with primary resistance to HER2-targeted therapy. The data presented by Zhang et al. and Sanz-Moreno et al. were consistent with this. This combination of evidence raises the possibility that pre-emptive treatment with trastuzumab and RANK-L inhibitor denosumab could prevent the development of resistance to HER2-directed therapy.

Taking these data into consideration, it is possible that a synergistic interaction between denosumab and trastuzumab may have played a role in the long-term progression-free survival of our three patients. Like chemotherapy, denosumab may help prevent resistance to HER2-targeted therapy. For instance, resistance to trastuzumab develops in 20-50% of cases when chemotherapy is given simultaneously, compared to 66-88% of cases when trastuzumab is used alone [19]. Possible mechanisms for drug synergism between trastuzumab and denosumab include inhibition of HER2/RANK heterodimerization [15] and reduced activation of nuclear factor kappa-Β (NF-κΒ) [17].

## Conclusions

Here, we presented three cases of bone-only metastatic breast cancer with unusually long progression-free survival, all measuring more than a decade. All patients were hormone receptor-positive and HER2-positive. All received endocrine therapy, HER2-directed therapy, and RANK-L inhibition treatment. All were able to tolerate over 10 years of HER2-directed therapy and continue without disease progression. We propose a possible synergy between RANK-L inhibitors, HER2-targeted therapy, and possibly endocrine therapy in metastatic breast cancer. At a pre-clinical level, this synergistic relationship appears to be due to the heterodimerization of RANK and HER2 receptors on cancer cells, as well as through interactions in their downstream signalling pathways, and perhaps prevents the development of resistance to HER2-directed therapy. However, the retrospective nature of this study limits generalizability. Further research is recommended to investigate this possible synergy in the context of stable bone-only metastatic disease. Likewise, further research is necessary to understand the adverse effect of foot fractures in the first case, possibly related to prolonged trastuzumab or denosumab usage. Finally, the role of anti-oestrogen therapies on the hypothesized synergism between anti-HER2 therapy and RANK-L inhibitors cannot be commented upon, as all three of our patients were receiving different endocrine therapies.
